# 3-Hydroxy­pyridinium hydrogen chloranilate monohydrate

**DOI:** 10.1107/S1600536809046844

**Published:** 2009-11-11

**Authors:** Kazuma Gotoh, Hiroyuki Ishida

**Affiliations:** a Department of Chemistry, Faculty of Science, Okayama University, Okayama 700-8530, Japan

## Abstract

In the title salt hydrate, C_5_H_6_NO^+^·C_6_HCl_2_O_4_^−^·H_2_O, the three components are held together by O—H⋯O and N—H⋯O hydrogen bonds, as well as by C—H⋯O contacts, forming a double-tape structure along the *c* axis. Within each tape, the pyridinium ring and the chloranilate ring are almost coplanar, forming a dihedral angle of 2.35 (7)°.

## Related literature

For related structures, see, for example: Gotoh *et al.* (2009*a* ▶,*b* ▶); Gotoh & Ishida (2009 ▶).
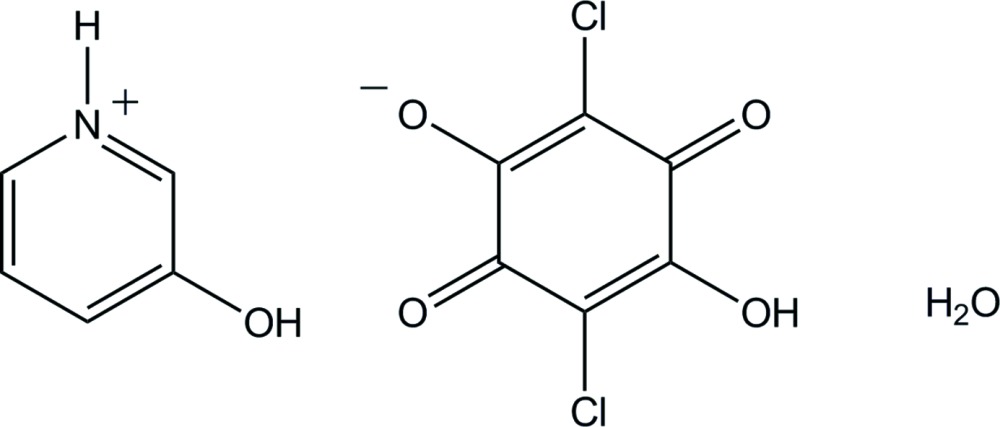


## Experimental

### 

#### Crystal data

C_5_H_6_NO^+^·C_6_HCl_2_O_4_^−^·H_2_O
*M* *_r_* = 322.10
Triclinic, 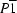

*a* = 7.4893 (13) Å
*b* = 9.6650 (17) Å
*c* = 9.9305 (17) Å
α = 88.129 (5)°
β = 68.404 (6)°
γ = 67.980 (4)°
*V* = 614.95 (18) Å^3^
*Z* = 2
Mo *K*α radiation
μ = 0.55 mm^−1^
*T* = 180 K
0.20 × 0.15 × 0.05 mm

#### Data collection

Rigaku R-AXIS RAPID-II diffractometer
Absorption correction: numerical (**ABSCOR**; Higashi, 1999 ▶) *T*_min_ = 0.907, *T*_max_ = 0.973
12237 measured reflections
3572 independent reflections
2952 reflections with *I* > 2σ(*I*)
*R*_int_ = 0.025

#### Refinement

*R*[*F* ^2^ > 2σ(*F* ^2^)] = 0.030
*wR*(*F* ^2^) = 0.088
*S* = 1.07
3572 reflections
201 parameters
H atoms treated by a mixture of independent and constrained refinement
Δρ_max_ = 0.60 e Å^−3^
Δρ_min_ = −0.29 e Å^−3^

### 

Data collection: *PROCESS-AUTO* (Rigaku/MSC, 2004 ▶); cell refinement: *PROCESS-AUTO*; data reduction: *CrystalStructure* (Rigaku/MSC, 2004 ▶); program(s) used to solve structure: *SIR92* (Altomare *et al.*, 1994 ▶); program(s) used to refine structure: *SHELXL97* (Sheldrick, 2008 ▶); molecular graphics: *ORTEP-3* (Farrugia, 1997 ▶); software used to prepare material for publication: *CrystalStructure* and *PLATON* (Spek, 2009 ▶).

## Supplementary Material

Crystal structure: contains datablocks global, I. DOI: 10.1107/S1600536809046844/tk2567sup1.cif

Structure factors: contains datablocks I. DOI: 10.1107/S1600536809046844/tk2567Isup2.hkl

Additional supplementary materials:  crystallographic information; 3D view; checkCIF report

## Figures and Tables

**Table 1:** Hydrogen-bond geometry (Å, °)

*D*—H⋯*A*	*D*—H	H⋯*A*	*D*⋯*A*	*D*—H⋯*A*
N1—H1⋯O2^i^	0.911 (18)	1.867 (18)	2.7461 (17)	161.4 (18)
O4—H4⋯O1	0.77 (3)	2.21 (3)	2.6348 (16)	115 (2)
O4—H4⋯O6	0.77 (3)	2.04 (3)	2.7187 (17)	147 (3)
O5—H5⋯O1	0.85 (3)	1.80 (3)	2.6474 (17)	172 (3)
O6—H6*A*⋯O2^i^	0.80 (3)	2.21 (3)	2.8959 (18)	144 (3)
O6—H6*A*⋯O3^i^	0.80 (3)	2.50 (3)	3.1220 (17)	136 (3)
O6—H6*B*⋯O1^ii^	0.84 (4)	2.11 (3)	2.9281 (18)	164 (3)
C7—H7⋯O6	0.95	2.59	3.484 (2)	157
C9—H9⋯O4^iii^	0.95	2.40	3.3084 (18)	160
C10—H10⋯O3^iv^	0.95	2.38	3.163 (2)	140

Symmetry codes: (i) 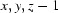; (ii) 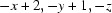; (iii) 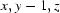; (iv) 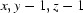.

**Fig. 1.**
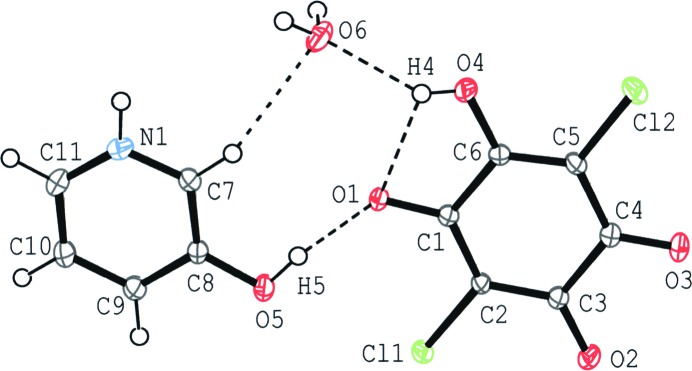
The molecular structures of the constituents in (I), with the atom-labeling. Displacement ellipsoids of non-H atoms are drawn at the 50% probability level. The dashed lines indicate O—H···O hydrogen bonds and C—H···O contacts.

**Fig. 2.**
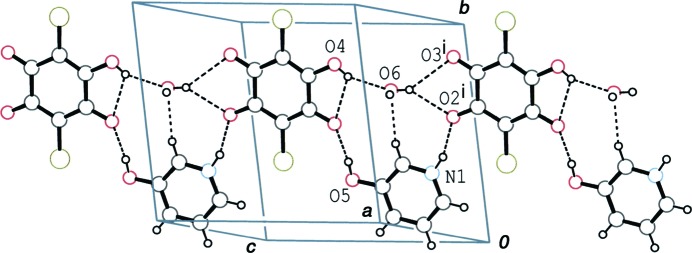
A partial packing diagram for (I), showing a molecular tape running along the c axis. The dashed lines indicate O—H···O and N—H···O hydrogen bonds, and C—H···O contacts.

**Fig. 3.**
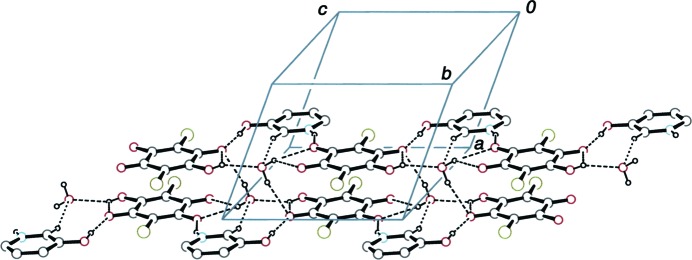
A partial packing diagram for (I), showing a double-tape structure running along the c axis. The dashed lines indicate O—H···O and N—H···O hydrogen bonds, and C—H···O contacts. H atoms not involved in the hydrogen bonds have been omitted.

## supplementary crystallographic information

### Comment

The title salt hydrate, C_5_H_6_NO^+^.C_6_HCl_2_O_4_^-^.H_2_O, (I), was prepared in order to extend our study on *D*—H···*A* hydrogen bonding (*D* = N, O, or C; *A* = N, O or Cl) in substituted-pyridine – chloranilic acid (systematic name: 2,5-dichloro-3,6-dihydroxy-1,4-benzoquinone) systems (Gotoh & Ishida, 2009; Gotoh *et al.*, 2009*a*,*b*).

In (I), the three components are held together by O—H···O and N—H···O hydrogen bonds, as well as C—H···O contacts (Fig. 1 and Table 1) forming a double-tape structure along the *c* direction. The connections between individual tapes, Fig. 2, are accomplished via O_water_–H···O hydrogen bonds, Fig. 3. Within each tape, the pyridinium N1/C7–C11 and the anion C1–C6 rings are almost coplanar, with a dihedral angle of 2.35 (7)° between them. A π–π interaction between the anion rings is also present within the double-tape structure; the centroid-centroid distance [*Cg*1···*Cg*1^iii^; symmetry code: (iii) -*x* + 2, -*y* + 1, -*z* + 1] is 3.6729 (11) Å and the inter-planar separation is 3.2656 (6) Å. The double-tapes are connected by C—H···O contacts, resulting in a layer parallel to the (100) plane, Table 1.

### Experimental

Single crystals were obtained by slow evaporation from a methanol solution (150 ml) of chloranilic acid (350 mg) and 3-hydroxypyridine (160 mg) at room temperature.

### Refinement

All H atoms were found in a difference Fourier map and O- and N-bound H atoms were refined isotropically. The refined O—H and N—H bond lengths are given in Table 1. C-bound H atoms were positioned geometrically (C—H = 0.95 Å) and refined as riding, with *U*_iso_(H) = 1.2*U*_eq_(C).

### Crystal data

**Table:**

C_5_H_6_NO^+^·C_6_HCl_2_O_4_^−^·H_2_O	*Z* = 2
*M**_r_* = 322.10	*F*(000) = 328.00
Triclinic, *P*1	*D*_x_ = 1.739 Mg m^−^^3^
Hall symbol: -P 1	Mo *K*α radiation, λ = 0.71075 Å
*a* = 7.4893 (13) Å	Cell parameters from 10001 reflections
*b* = 9.6650 (17) Å	θ = 3.0–30.1°
*c* = 9.9305 (17) Å	µ = 0.55 mm^−^^1^
α = 88.129 (5)°	*T* = 180 K
β = 68.404 (6)°	Block, brown
γ = 67.980 (4)°	0.20 × 0.15 × 0.05 mm
*V* = 614.95 (18) Å^3^	

### Data collection

**Table:**

Rigaku R-AXIS RAPID-II diffractometer	2952 reflections with *I* > 2σ(*I*)
Detector resolution: 10.00 pixels mm^-1^	*R*_int_ = 0.025
ω scans	θ_max_ = 30.0°
Absorption correction: numerical (*ABSCOR*; Higashi, 1999)	*h* = −10→10
*T*_min_ = 0.907, *T*_max_ = 0.973	*k* = −13→13
12237 measured reflections	*l* = −13→13
3572 independent reflections	

### Refinement

**Table:**

Refinement on *F*^2^	Primary atom site location: structure-invariant direct methods
Least-squares matrix: full	Secondary atom site location: difference Fourier map
*R*[*F*^2^ > 2σ(*F*^2^)] = 0.030	Hydrogen site location: inferred from neighbouring sites
*wR*(*F*^2^) = 0.088	H atoms treated by a mixture of independent and constrained refinement
*S* = 1.07	*w* = 1/[σ^2^(*F*_o_^2^) + (0.0481*P*)^2^ + 0.2238*P*] where *P* = (*F*_o_^2^ + 2*F*_c_^2^)/3
3572 reflections	(Δ/σ)_max_ < 0.001
201 parameters	Δρ_max_ = 0.60 e Å^−^^3^
0 restraints	Δρ_min_ = −0.29 e Å^−^^3^

### Special details

**Table:**

Geometry. All e.s.d.'s (except the e.s.d. in the dihedral angle between two l.s. planes) are estimated using the full covariance matrix. The cell e.s.d.'s are taken into account individually in the estimation of e.s.d.'s in distances, angles and torsion angles; correlations between e.s.d.'s in cell parameters are only used when they are defined by crystal symmetry. An approximate (isotropic) treatment of cell e.s.d.'s is used for estimating e.s.d.'s involving l.s. planes.
Refinement. Refinement of *F*^2^ against ALL reflections. The weighted *R*-factor *wR* and goodness of fit *S* are based on *F*^2^, conventional *R*-factors *R* are based on *F*, with *F* set to zero for negative *F*^2^. The threshold expression of *F*^2^ > σ(*F*^2^) is used only for calculating *R*-factors(gt) *etc*. and is not relevant to the choice of reflections for refinement. *R*-factors based on *F*^2^ are statistically about twice as large as those based on *F*, and *R*- factors based on ALL data will be even larger.

### Fractional atomic coordinates and isotropic or equivalent isotropic displacement parameters (Å^2^)

**Table:**

	*x*	*y*	*z*	*U*_iso_*/*U*_eq_	
Cl1	0.74975 (5)	0.30013 (3)	0.52240 (3)	0.01897 (9)	
Cl2	0.76493 (5)	0.94942 (3)	0.44706 (4)	0.02295 (10)	
O1	0.77201 (16)	0.47186 (11)	0.26178 (10)	0.0198 (2)	
O2	0.72724 (17)	0.51208 (11)	0.74674 (10)	0.0226 (2)	
O3	0.73261 (17)	0.78687 (12)	0.71214 (11)	0.0244 (2)	
O4	0.76878 (18)	0.74463 (12)	0.23264 (11)	0.0238 (2)	
O5	0.7211 (2)	0.21895 (12)	0.23481 (11)	0.0277 (2)	
O6	0.8140 (2)	0.62351 (14)	−0.02703 (12)	0.0284 (2)	
N1	0.7338 (2)	0.25226 (14)	−0.13073 (13)	0.0229 (2)	
C1	0.76153 (19)	0.53710 (14)	0.37347 (13)	0.0153 (2)	
C2	0.7509 (2)	0.47845 (13)	0.50577 (13)	0.0152 (2)	
C3	0.7388 (2)	0.55870 (14)	0.62593 (13)	0.0162 (2)	
C4	0.7419 (2)	0.71690 (14)	0.60863 (14)	0.0173 (2)	
C5	0.7570 (2)	0.77458 (14)	0.46900 (14)	0.0168 (2)	
C6	0.7618 (2)	0.69224 (14)	0.35935 (14)	0.0165 (2)	
C7	0.7293 (2)	0.29123 (15)	−0.00035 (15)	0.0199 (3)	
H7	0.7272	0.3870	0.0215	0.024*	
C8	0.7279 (2)	0.18979 (15)	0.10182 (14)	0.0190 (3)	
C9	0.7317 (2)	0.05028 (15)	0.06603 (15)	0.0222 (3)	
H9	0.7297	−0.0203	0.1348	0.027*	
C10	0.7383 (2)	0.01496 (16)	−0.06979 (16)	0.0239 (3)	
H10	0.7428	−0.0805	−0.0954	0.029*	
C11	0.7382 (2)	0.11952 (17)	−0.16830 (15)	0.0252 (3)	
H11	0.7413	0.0970	−0.2617	0.030*	
H1	0.731 (3)	0.328 (2)	−0.188 (2)	0.041 (6)*	
H6A	0.759 (4)	0.633 (3)	−0.084 (3)	0.067 (8)*	
H6B	0.941 (5)	0.594 (3)	−0.081 (3)	0.061 (8)*	
H4	0.762 (4)	0.690 (3)	0.182 (3)	0.063 (8)*	
H5	0.739 (4)	0.301 (3)	0.235 (3)	0.060 (7)*	

### Atomic displacement parameters (Å^2^)

**Table:**

	*U*^11^	*U*^22^	*U*^33^	*U*^12^	*U*^13^	*U*^23^
Cl1	0.02939 (17)	0.01539 (14)	0.01838 (15)	−0.01195 (12)	−0.01284 (12)	0.00521 (11)
Cl2	0.03172 (18)	0.01444 (15)	0.02679 (18)	−0.01173 (13)	−0.01295 (14)	0.00362 (11)
O1	0.0324 (5)	0.0193 (4)	0.0138 (4)	−0.0142 (4)	−0.0113 (4)	0.0028 (3)
O2	0.0366 (6)	0.0214 (5)	0.0159 (4)	−0.0141 (4)	−0.0140 (4)	0.0051 (4)
O3	0.0360 (6)	0.0239 (5)	0.0181 (5)	−0.0149 (4)	−0.0120 (4)	−0.0016 (4)
O4	0.0443 (6)	0.0186 (5)	0.0171 (5)	−0.0168 (4)	−0.0166 (4)	0.0069 (4)
O5	0.0531 (7)	0.0237 (5)	0.0208 (5)	−0.0232 (5)	−0.0218 (5)	0.0071 (4)
O6	0.0357 (6)	0.0369 (6)	0.0159 (5)	−0.0149 (5)	−0.0124 (5)	0.0023 (4)
N1	0.0314 (6)	0.0236 (6)	0.0174 (5)	−0.0136 (5)	−0.0106 (5)	0.0073 (4)
C1	0.0189 (6)	0.0146 (5)	0.0145 (5)	−0.0077 (4)	−0.0076 (4)	0.0027 (4)
C2	0.0212 (6)	0.0130 (5)	0.0145 (6)	−0.0081 (4)	−0.0088 (5)	0.0029 (4)
C3	0.0192 (6)	0.0162 (5)	0.0152 (6)	−0.0077 (5)	−0.0079 (5)	0.0023 (4)
C4	0.0204 (6)	0.0180 (6)	0.0157 (6)	−0.0086 (5)	−0.0080 (5)	0.0011 (4)
C5	0.0218 (6)	0.0128 (5)	0.0185 (6)	−0.0086 (5)	−0.0088 (5)	0.0030 (4)
C6	0.0221 (6)	0.0152 (5)	0.0150 (6)	−0.0091 (5)	−0.0086 (5)	0.0043 (4)
C7	0.0272 (7)	0.0171 (6)	0.0191 (6)	−0.0114 (5)	−0.0101 (5)	0.0034 (5)
C8	0.0261 (7)	0.0181 (6)	0.0170 (6)	−0.0111 (5)	−0.0103 (5)	0.0026 (5)
C9	0.0340 (7)	0.0181 (6)	0.0202 (6)	−0.0134 (5)	−0.0134 (6)	0.0049 (5)
C10	0.0335 (7)	0.0206 (6)	0.0219 (7)	−0.0137 (6)	−0.0118 (6)	0.0002 (5)
C11	0.0344 (8)	0.0295 (7)	0.0162 (6)	−0.0157 (6)	−0.0113 (5)	0.0023 (5)

### Geometric parameters (Å, °)

**Table:**

Cl1—C2	1.7289 (13)	C1—C2	1.4007 (17)
Cl2—C5	1.7172 (13)	C1—C6	1.5020 (17)
O1—C1	1.2564 (15)	C2—C3	1.4006 (17)
O2—C3	1.2519 (15)	C3—C4	1.5412 (18)
O3—C4	1.2149 (16)	C4—C5	1.4587 (18)
O4—C6	1.3313 (15)	C5—C6	1.3514 (18)
O4—H4	0.76 (3)	C7—C8	1.3877 (18)
O5—C8	1.3381 (16)	C7—H7	0.9500
O5—H5	0.85 (3)	C8—C9	1.3930 (18)
O6—H6A	0.80 (3)	C9—C10	1.3808 (19)
O6—H6B	0.84 (3)	C9—H9	0.9500
N1—C11	1.3330 (19)	C10—C11	1.383 (2)
N1—C7	1.3452 (18)	C10—H10	0.9500
N1—H1	0.91 (2)	C11—H11	0.9500
			
C6—O4—H4	111 (2)	C4—C5—Cl2	119.01 (9)
C8—O5—H5	106.0 (18)	O4—C6—C5	121.39 (11)
H6A—O6—H6B	103 (3)	O4—C6—C1	116.69 (11)
C11—N1—C7	123.32 (12)	C5—C6—C1	121.91 (11)
C11—N1—H1	125.1 (14)	N1—C7—C8	119.14 (12)
C7—N1—H1	111.6 (14)	N1—C7—H7	120.4
O1—C1—C2	126.22 (11)	C8—C7—H7	120.4
O1—C1—C6	115.16 (11)	O5—C8—C7	123.49 (12)
C2—C1—C6	118.62 (11)	O5—C8—C9	117.59 (12)
C3—C2—C1	122.83 (11)	C7—C8—C9	118.92 (12)
C3—C2—Cl1	118.48 (9)	C10—C9—C8	119.78 (13)
C1—C2—Cl1	118.68 (9)	C10—C9—H9	120.1
O2—C3—C2	125.37 (12)	C8—C9—H9	120.1
O2—C3—C4	116.88 (11)	C9—C10—C11	119.55 (13)
C2—C3—C4	117.75 (11)	C9—C10—H10	120.2
O3—C4—C5	123.42 (12)	C11—C10—H10	120.2
O3—C4—C3	118.12 (11)	N1—C11—C10	119.29 (13)
C5—C4—C3	118.46 (10)	N1—C11—H11	120.4
C6—C5—C4	120.38 (11)	C10—C11—H11	120.4
C6—C5—Cl2	120.61 (10)		
			
O1—C1—C2—C3	−179.78 (13)	C4—C5—C6—O4	177.85 (12)
C6—C1—C2—C3	0.53 (19)	Cl2—C5—C6—O4	−1.29 (19)
O1—C1—C2—Cl1	−0.50 (19)	C4—C5—C6—C1	−2.7 (2)
C6—C1—C2—Cl1	179.80 (9)	Cl2—C5—C6—C1	178.13 (10)
C1—C2—C3—O2	179.38 (13)	O1—C1—C6—O4	1.29 (17)
Cl1—C2—C3—O2	0.10 (19)	C2—C1—C6—O4	−178.98 (12)
C1—C2—C3—C4	−1.25 (19)	O1—C1—C6—C5	−178.16 (12)
Cl1—C2—C3—C4	179.47 (9)	C2—C1—C6—C5	1.57 (19)
O2—C3—C4—O3	−0.19 (19)	C11—N1—C7—C8	−0.4 (2)
C2—C3—C4—O3	−179.62 (12)	N1—C7—C8—O5	−179.23 (13)
O2—C3—C4—C5	179.52 (12)	N1—C7—C8—C9	0.2 (2)
C2—C3—C4—C5	0.09 (18)	O5—C8—C9—C10	179.88 (14)
O3—C4—C5—C6	−178.41 (13)	C7—C8—C9—C10	0.5 (2)
C3—C4—C5—C6	1.89 (19)	C8—C9—C10—C11	−0.9 (2)
O3—C4—C5—Cl2	0.74 (19)	C7—N1—C11—C10	0.0 (2)
C3—C4—C5—Cl2	−178.96 (9)	C9—C10—C11—N1	0.6 (2)

### Hydrogen-bond geometry (Å, °)

**Table:**

*D*—H···*A*	*D*—H	H···*A*	*D*···*A*	*D*—H···*A*
N1—H1···O2^i^	0.911 (18)	1.867 (18)	2.7461 (17)	161.4 (18)
O4—H4···O1	0.77 (3)	2.21 (3)	2.6348 (16)	115 (2)
O4—H4···O6	0.77 (3)	2.04 (3)	2.7187 (17)	147 (3)
O5—H5···O1	0.85 (3)	1.80 (3)	2.6474 (17)	172 (3)
O6—H6A···O2^i^	0.80 (3)	2.21 (3)	2.8959 (18)	144 (3)
O6—H6A···O3^i^	0.80 (3)	2.50 (3)	3.1220 (17)	136 (3)
O6—H6B···O1^ii^	0.84 (4)	2.11 (3)	2.9281 (18)	164 (3)
C7—H7···O6	0.95	2.59	3.484 (2)	157
C9—H9···O4^iii^	0.95	2.40	3.3084 (18)	160
C10—H10···O3^iv^	0.95	2.38	3.163 (2)	140

Symmetry codes: (i) *x*, *y*, *z*−1; (ii) −*x*+2, −*y*+1, −*z*; (iii) *x*, *y*−1, *z*; (iv) *x*, *y*−1, *z*−1.
